# Supplementary material for: SMN deficiency perturbs monoamine neurotransmitter metabolism in spinal muscular atrophy
Source: Commun Biol. 2023 Nov 13;6:1155. doi: 10.1038/s42003-023-05543-1 (PMC10643621; doi:10.1038/s42003-023-05543-1)
Supplement: Supplementary file 2 — Supplementary Information [file 42003_2023_5543_MOESM2_ESM.pdf]

Supplementary information for

SMN deficiency perturbs monoamine neurotransmitter metabolism in spinal muscular atrophy

Short title: Norepinephrine dysmetabolism in SMA.

Valeria Valsecchi[1,†], Francesco Errico[2,3,†], Valentina Bassareo[4,†], Carmen Marino[5,†], Tommaso Nuzzo[3,6], Paola Brancaccio[1], Giusy Laudati[1], Antonella Casamassa[7], Manuela Grimaldi[5], Adele D'Amico[8], Manolo Carta[4], Anna Maria D'Ursi[5], Giuseppe Pignataro[1], Enrico Bertini[8], Alessandro Usiello[3,6,*]

[1]Division of Pharmacology, Department of Neuroscience, Reproductive and Dentistry Sciences, School of Medicine, University of Naples "Federico II", 80131 Naples, Italy; [2]Department of Agricultural Sciences, University of Naples "Federico II", 80055, Portici, Italy; [3]Laboratory of Translational Neuroscience, Ceinge Biotecnologie Avanzate, 80145, Naples, Italy; [4]Department of Biomedical Sciences, University of Cagliari, 09042, Monserrato, Italy; [5]Department of Pharmacy, University of Salerno, 84084, Fisciano, Salerno, Italy; [6]Department of Environmental, Biological and Pharmaceutical Science and Technologies, Università degli Studi della Campania "Luigi Vanvitelli", 81100, Caserta, Italy; [7] IRCCS Synlab SDN, 80143, Naples, Italy; [8]Unit of Neuromuscular and Neurodegenerative Disorders, Dept. Neurosciences, Bambino Gesù Children's Hospital IRCCS, 00163, Rome, Italy.

[†] These authors contributed equally to this work.

* Corresponding Author:
Alessandro Usiello, PhD: Department of Environmental, Biological and Pharmaceutical Sciences and Technologies, University of Campania "Luigi Vanvitelli", Via A. Vivaldi, 43, 81100 Caserta, Italy, and CEINGE Biotecnologie Avanzate, Naples, Italy; Phone: +39 0813737879, email: usiello@ceinge.unina.it.

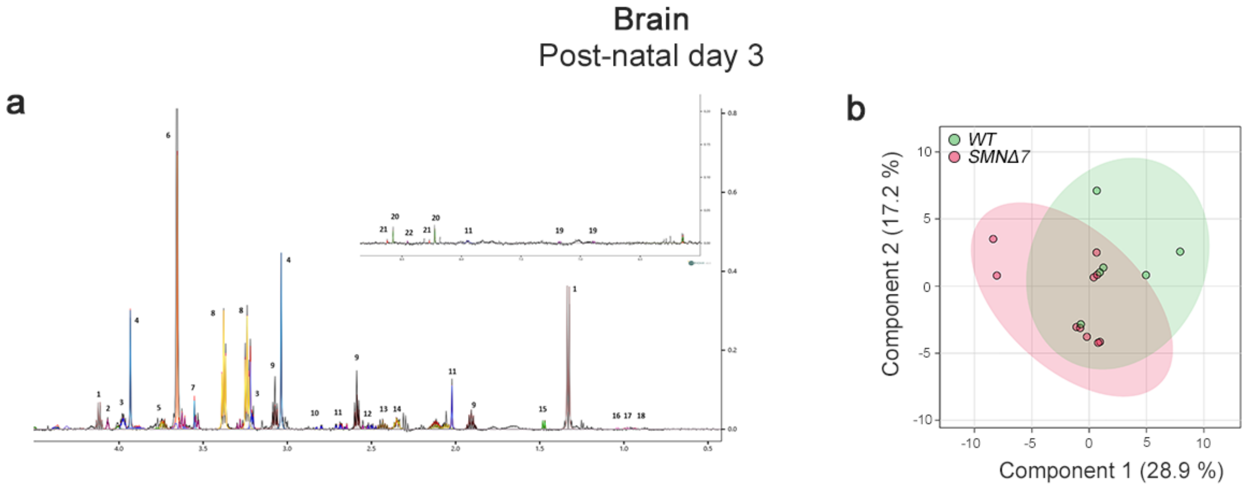

Supplementary Figure 1. *Untargeted NMR-based metabolome analysis in the SMNΔ7 mouse brain at post-natal day 3*. Representative 1D ^{1}H NOESY spectrum of brain polar extracts. The spectrum on top is enlarged for better visualization. The spectrum is acquired at 600 MHz and T = 310 K. **(a)** Spectrum related to P3 brains polar extract showed twenty-two metabolites identified and annotated as follow: 1: Lactate; 2: myo-Inositol; 3: O-Phosphoethanolamine; 4: Creatine; 5: sn-Glycero-3-phosphocholine; 6: Ethylene glycol; 7: Glycine; 8: Taurine; 9: 4-Aminobutyrate; 10: Aspartate; 11: N-Acetylaspartate; 12: Citrate; 13: Glutamine; 14: Glutamate; 15: Alanine; 16: Valine; 17: Isoleucine; 18: Leucine; 19: Tyrosine; 20: IMP; 21: ADP; 22: Formate **(b)** PLS-DA score scatter plots showing the metabolomic profile of brain polar extract from *SMNΔ7* (n=10) and wild type (WT) (n=6) mice at P3. The cluster analyses are reported in the Cartesian space that is described by the main components PC1: 28.9% and PC2:17.2%. PLS-DA was evaluated using cross-validation (CV) analysis. CV tests performed according to PLS-DA statistical protocol showed negative Q2 indices (-0.30,-0.40).

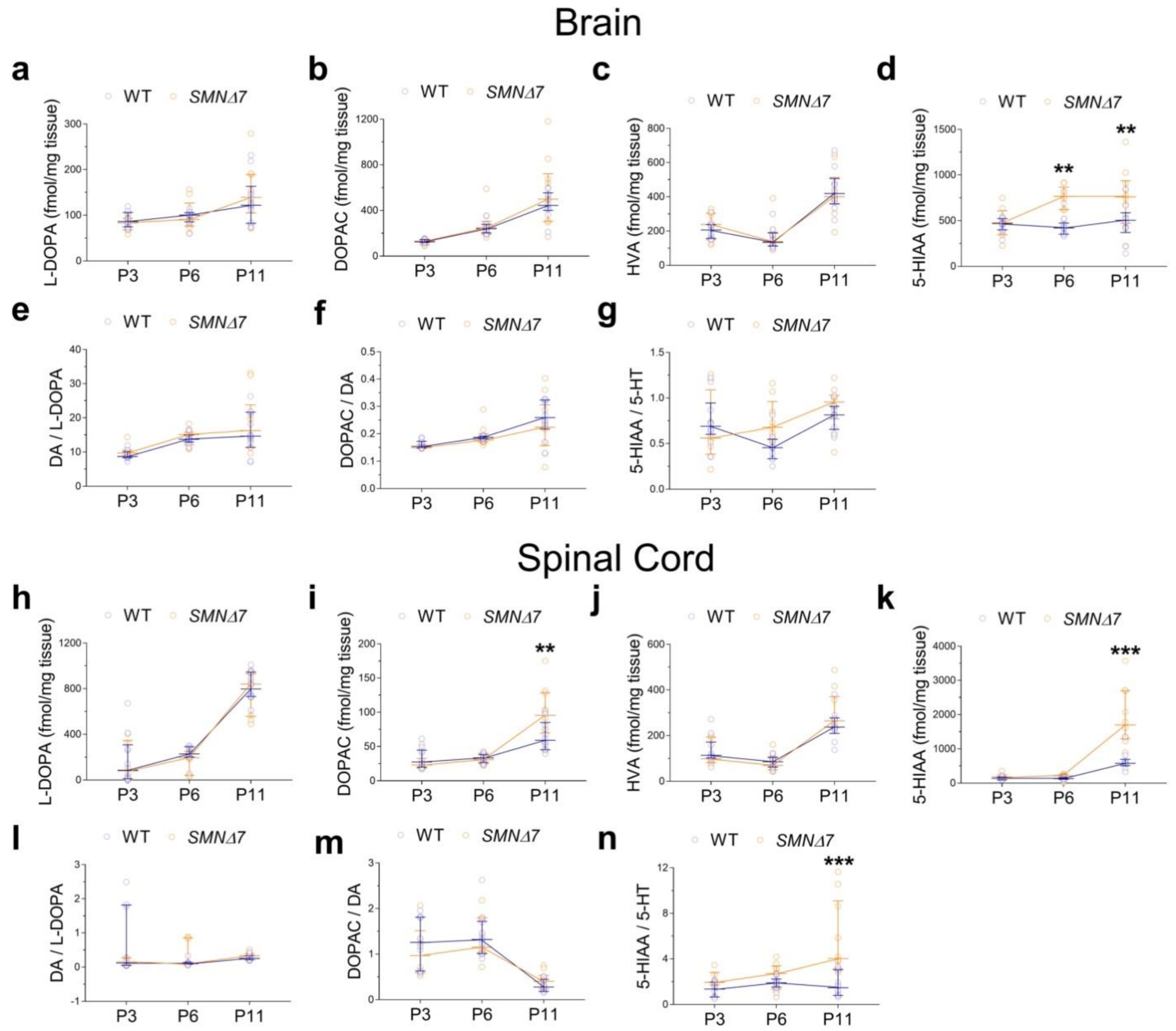

Supplementary Figure 2. *Analysis of monoamine metabolites levels in the brain and spinal cord of SMNΔ7 mice during the ontogeny*. Levels of **(a,h)** L-DOPA, **(b,i)** DOPAC, **(c,j)** HVA, **(d,k)** 5-HIAA, and **(e,l)** DA/L-DOPA, **(f,m)** DOPAC/DA, and **(g,n)** 5-HIAA/5-HT ratio in the **(a-g)** brain and **(h-n)** spinal cord of wild type (WT) and *SMNΔ7* mice at postnatal day 3 (P3), P6 and P11. The average amounts of monoamine detected were normalized for mg of wet tissue. Monoamine metabolites levels (fmol/mg tissue) are expressed as box and whisker plots representing median with interquartile range (IQR) and analzyed by two-way ANOVA, followed by multiple comparisons controlled by False Discovery Rate (FDR) method of Benjamini and Hochberg (B-H). $^{**}p < 0.01$, $^{***}p < 0.0001$, compared to age-matched WT mice.

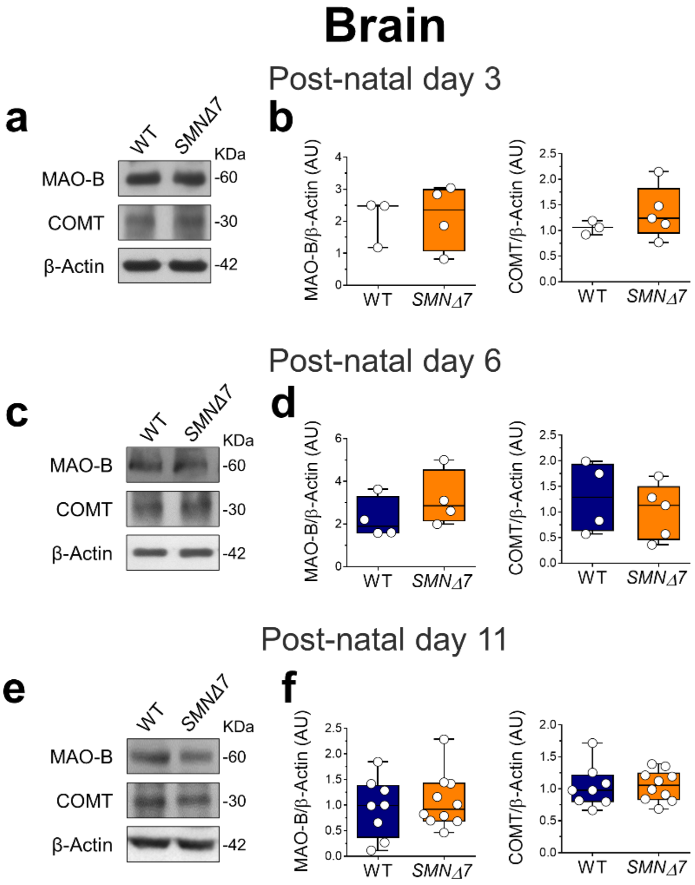

Supplementary Figure 3. *Analysis of MAO-B and COMT protein expression in the brain of SMNΔ7 mice during the ontogeny*. **(a,c,e)** Representative autoradiograms of brain lysates immunoblots of *SMNΔ7* and wild type (WT) mice at **(a)** post-natal day 3, **(c)** day 6 and **(e)** day 11. **(b,d,f)** Protein levels quantification of MAO-B (left panels) and COMT (right panels) protein levels at **(b)** post-natal day 3, **(d)** day 6 and **(f)** day 11. Data are normalized to β-Actin levels and shown as box and whisker plots representing median with interquartile range (IQR). Dots represent individual mice values.

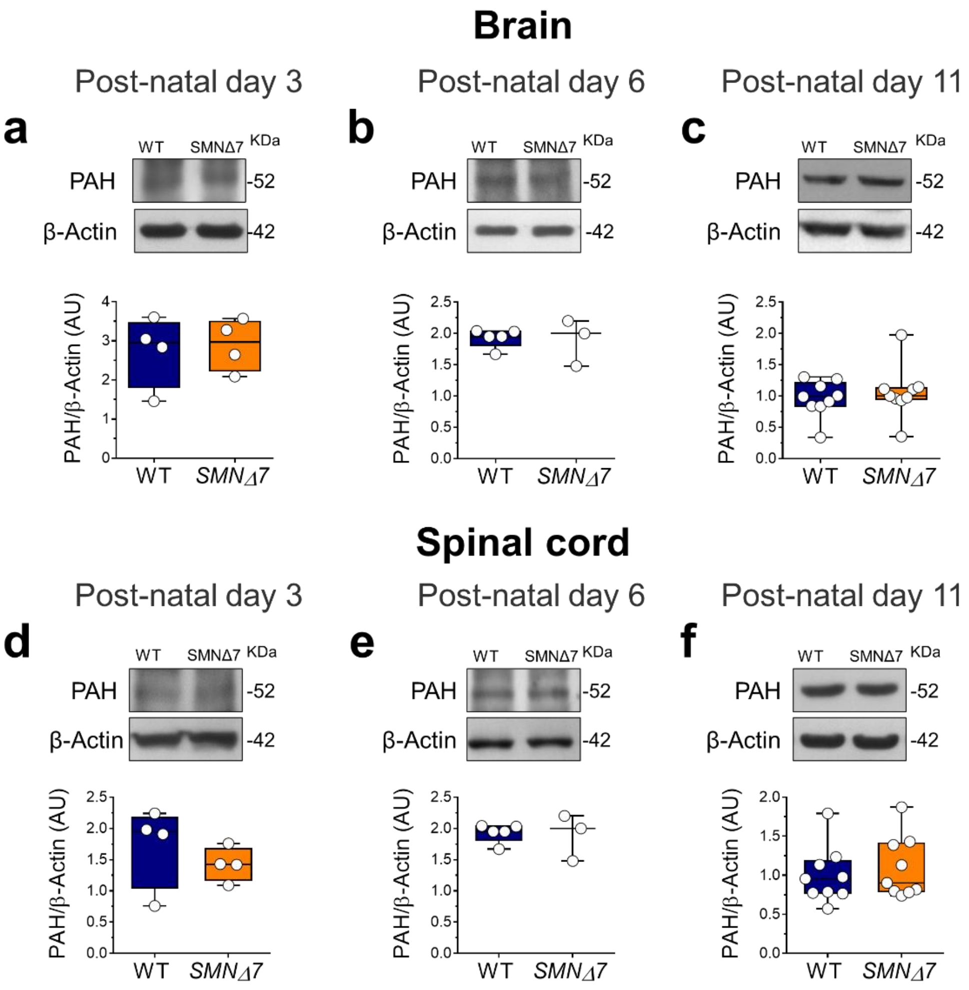

Supplementary Figure 4. *Analysis of phenylalanine hydroxylase in the brain and spinal cord of SMNΔ7 mice during the ontogeny*. Protein levels quantification of phenylalanine hydroxylase (PAH) in the **(a-c)** brain and **(d-f)** spinal cord of *SMNΔ7* and wild type (WT) mice at **(a,d)** post-natal day 3, **(b,e)** day 6 and **(c,f)** day 11. Representative autoradiograms of brain and spinal cord lysates immunoblot are shown above each graph. Data are normalized to β-Actin levels and shown as box and whisker plots representing median with interquartile range (IQR). Dots represent individual mice values.

Spinal cord

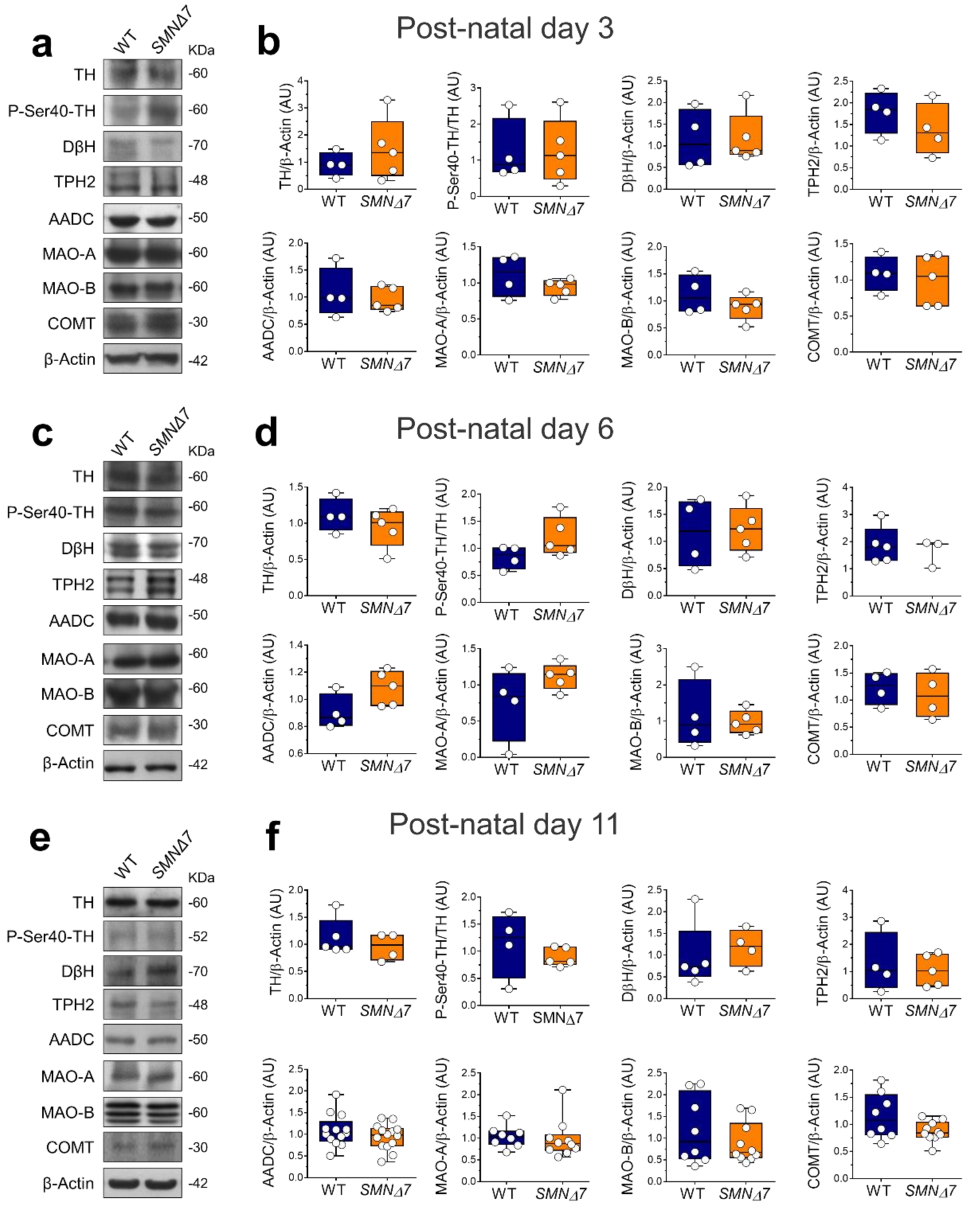

Supplementary Figure 5. *Analysis of monoamine-regulating enzymes in the brain of SMNΔ7 mice during the ontogeny*. **(a,c,e)** Representative autoradiograms of brain lysates immunoblots of *SMNΔ7* and wild type (WT) mice at **(a)** post-natal day 3, **(c)** day 6 and **(e)** day 11. **(b,d,f)** Protein levels quantification of Tyrosine hydroxylase (TH), phospho-Tyrosine hydroxylase at Ser-40 (P-Ser40-TH), Dopamine β hydroxylase (DβH), Tryptophan hydroxylase 2 (TPH2), Aromatic amino acid decarboxylase (AADC), Monoamine oxidase A (MAO-A), MAO-B and Catechol-*O*-methyltransferase (COMT) in *SMNΔ7* and WT mice at **(b)** post-natal day 3, **(d)** day 6 and **(f)** day 11. Data are normalized to β-Actin levels and shown as box and whisker plots representing the median with interquartile range (IQR). Dots represent individual mice values.

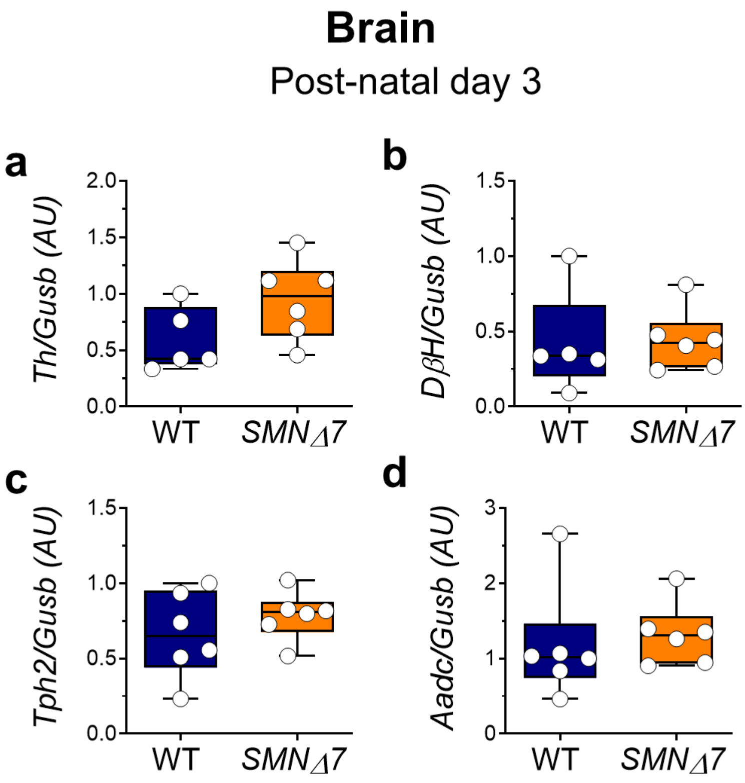

Supplementary Figure 6. *Analysis of Th, Dβh, Tph2 and Aadc mRNA levels in the brain of SMNΔ7 at post-natal day 3*. **(a-c)** Transcript levels of **(a)** Th, **(b)** *Dβh*, **(c)** *Tph2* and **(d)** *Aadc* in the brain of *SMNΔ7* and wild type (WT) mice at postnatal day 3. Data are expressed as the difference in threshold cycle ($2^{-\Delta\Delta Ct}$) between the target gene and the reference gene, *β-glucuronidase* (*Gusb*) (arbitrary units, AU), and shown as box and whisker plots representing median with interquartile range (IQR). Dots represent individual mice values.

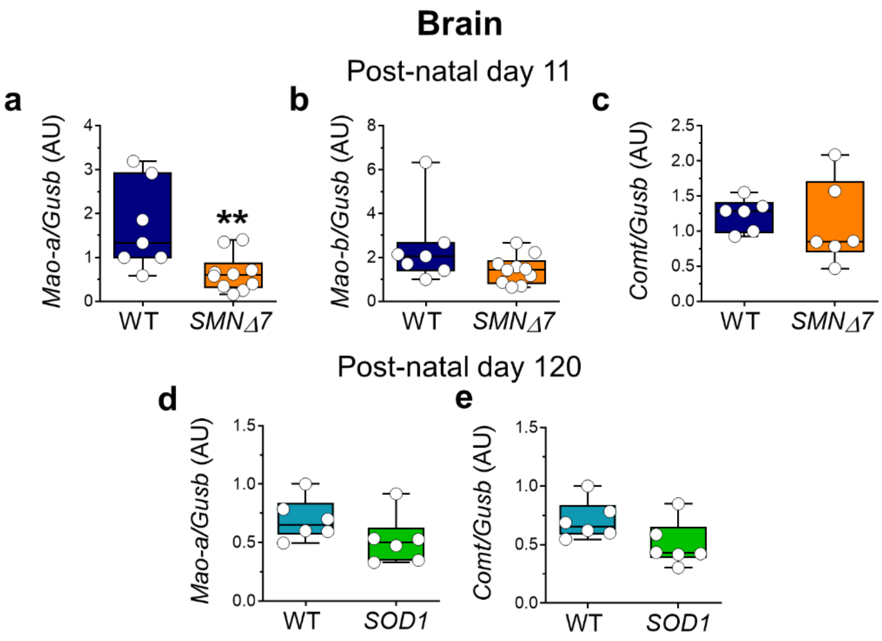

Supplementary Figure 7. *Analysis of Mao-a, Mao-b and Comt mRNA levels in the brain of SMNΔ7 and SOD1 mice*. **(a-c)** Transcript levels of **(a)** *Mao-a*, **(b)** *Mao-b* and **(c)** *Comt* in the brain of *SMNΔ7* and wild type (WT) mice at post-natal day 11. **(d,e)** Transcript levels of **(d)** *Mao-a* and **(e)** *Comt* in the brain of *SOD1* and wild type (WT) mice at post-natal day 120. Data are expressed as the difference in threshold cycle ($2^{-\Delta\Delta Ct}$) between the target gene and the reference gene, *β-glucuronidase* (*Gusb*) (arbitrary units, AU), and shown as box and whisker plots representing median with interquartile range (IQR). Dots represent individual mice values. **$p < 0.01$, unpaired *t* test.

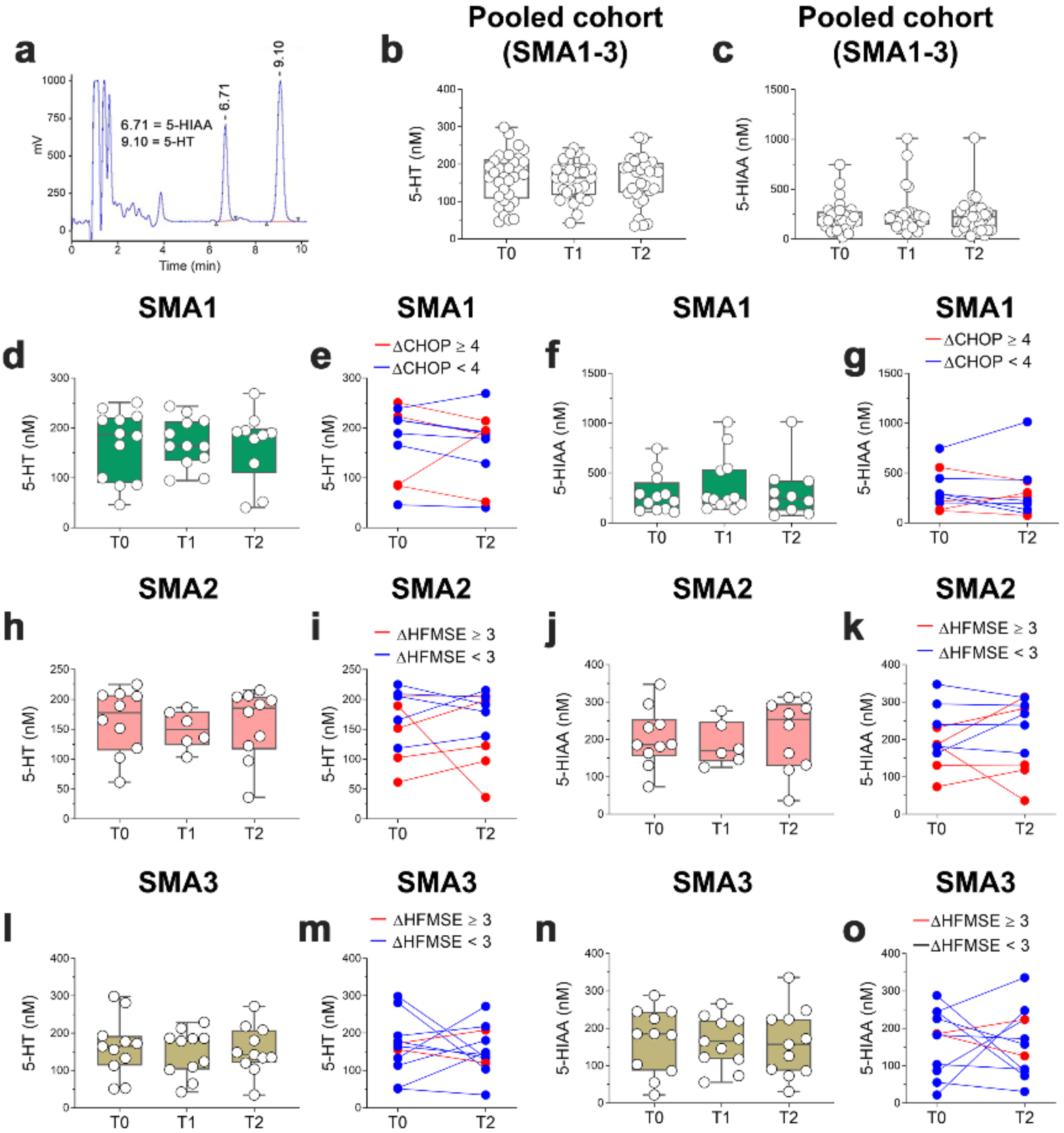

Supplementary Figure 8. *CSF levels of serotonin and 5-hydroxyindoleacetic acid in Nusinersen-treated SMA1, SMA2 and SMA3 patients.* (**a**) Representative CSF sample chromatogram of SMA patient showing serotonin (5-HT) and 5-hydroxyindoleacetic acid (5-HIAA) peaks and their related retention times. (**b,c**) Levels of **(b)** 5-HT and **(c)** 5-HIAA in the pooled cohort of SMA1, SMA2 and SMA3 patients (n=33) prior to treatment (T0) and at the time of the fourth (T1, loading phase) and the sixth (T2, maintenance phase) injection of Nusinersen. (**d,h,l,f,j,n**) Levels of **(d,hl)** 5-HT and **(f,j,n)** 5-HIAA in the CSF of **(d,f)** SMA1 (n=12), **(h,j)** SMA2 (n=10) and **(l,n)** SMA3 (n=11) patients at T0, T1 and T2. Data are shown as box and whisker plots representing the median with interquartile range (IQR). **(e,i,m,f,j,n)** *Spaghetti* plots representing variations of **(e,i,m)** 5-HT and **(f,j,n)** 5-HIAA levels in the CSF of **(e,g)** SMA1 (n=12), **(i,k)** SMA2 (n=10) and **(m,o)** SMA3 (n=11) patients between T0 and T2. Dots represent individual patients' values. Patients with ΔCHOP ≥ 4 or ΔHFMSE ≥ 3 are shown in red while patients with ΔCHOP < 4 or ΔHFMSE < 3 are shown in blue.

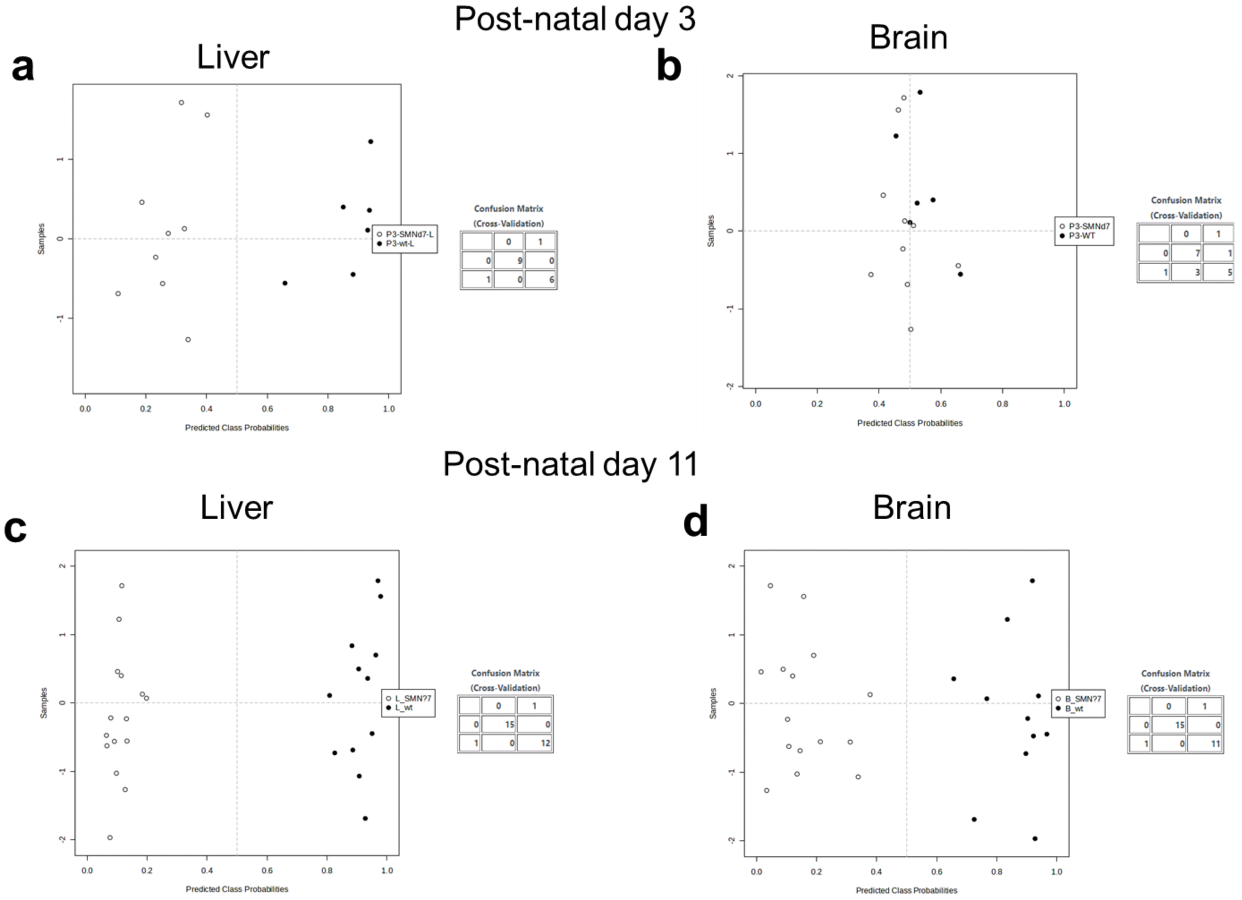

Supplementary Figure 9. *Graph related to class prediction conducted using Support vector Machine (SVM) method.* The prediction was made on the liver of *SMNΔ7* and WT mice at **(a)** P3 and **(c)** P11 and brain at **(b)** P3 and P11 **(d)** polar extracts. Each score plot corresponds to the relative confusion matrix (at the right of the graph), indicating the predicted sample number in each analyzed class.

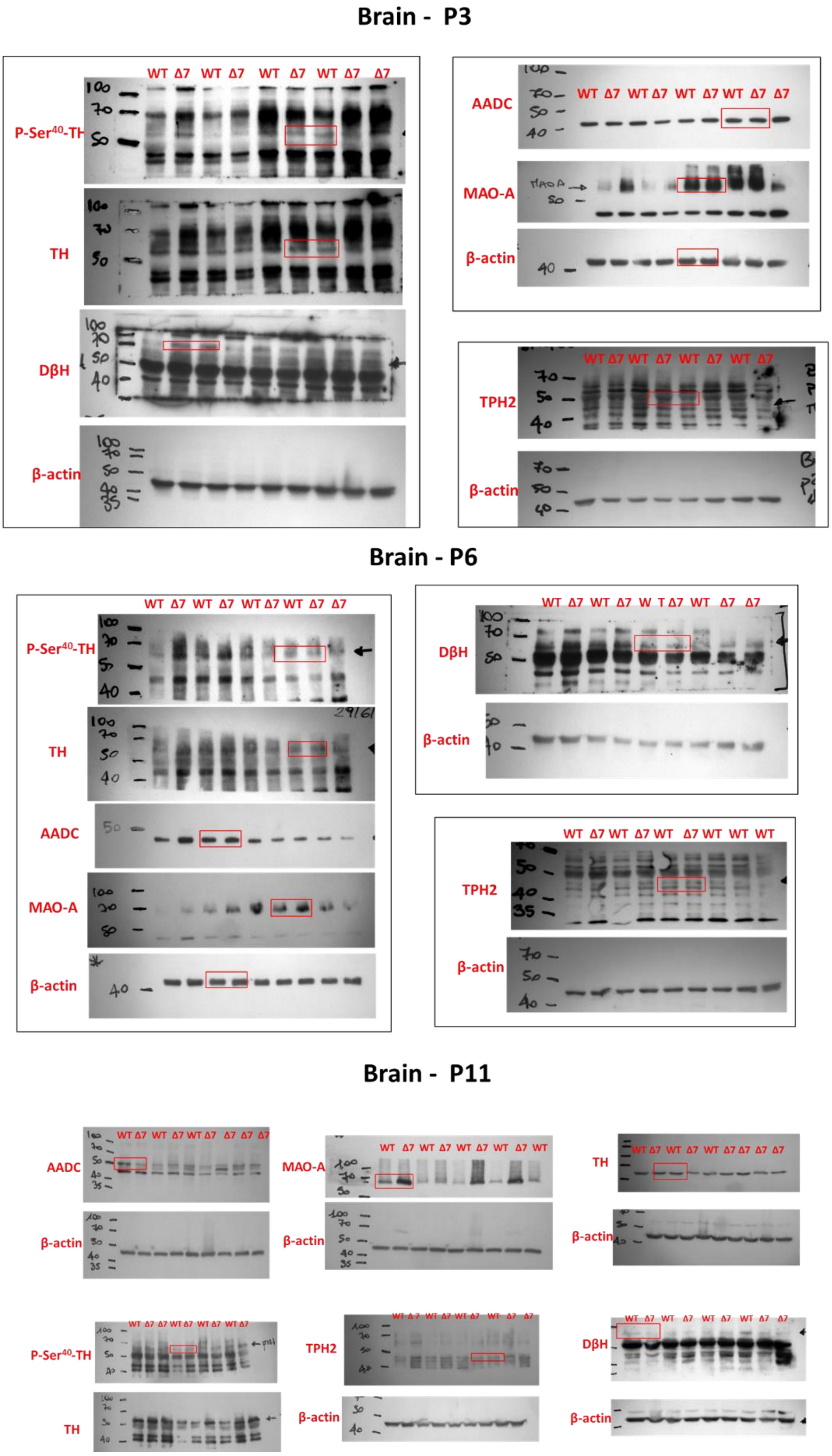

Supplementary Figure 10. Uncropped immunoblot images from Figure 5. Red boxes show the cropping limits used to generate the final panels.

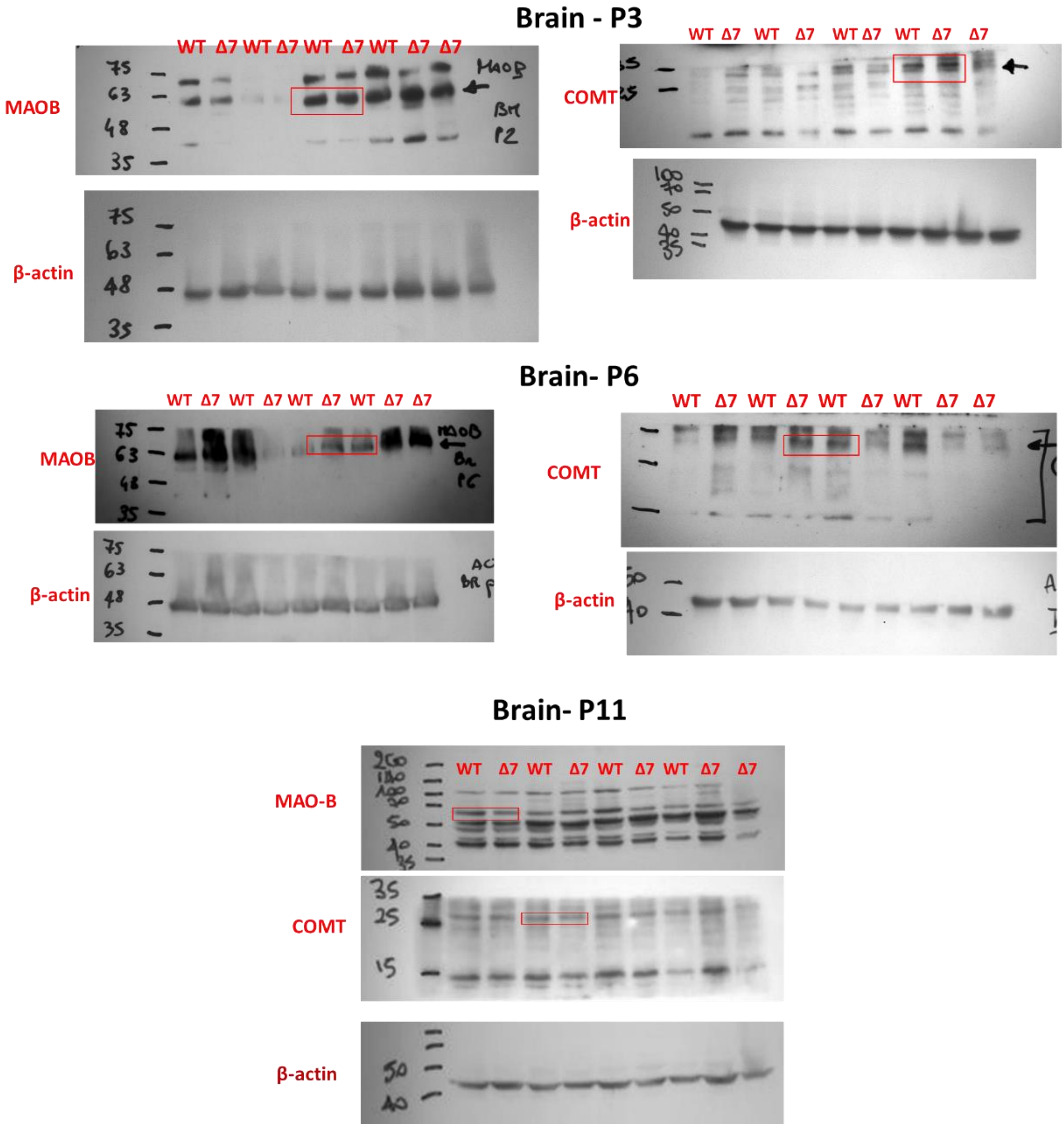

Supplementary Figure 11. Uncropped immunoblot images from Supplementary Figure 3. Red boxes show the cropping limits used to generate the final panels.

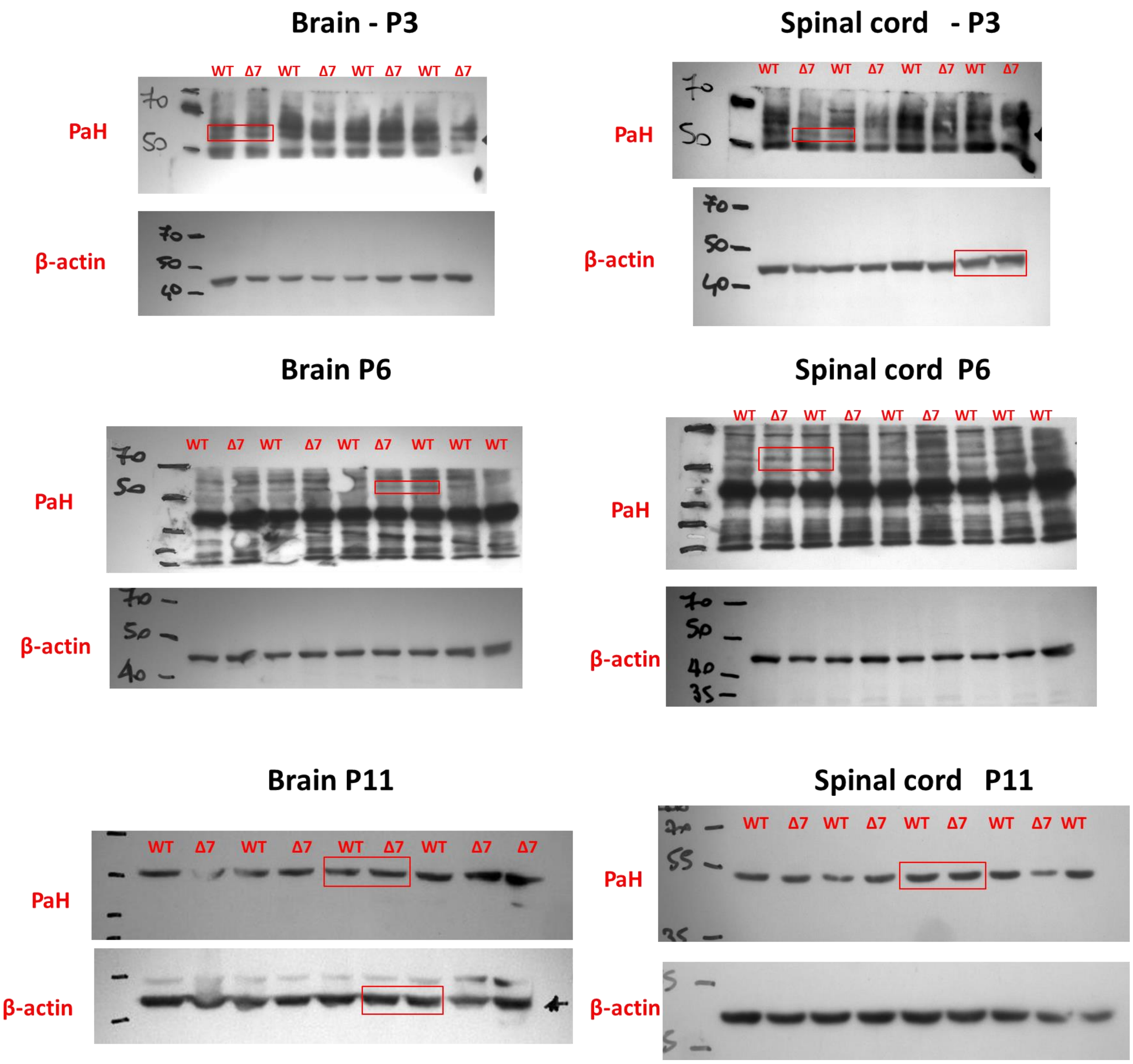

Supplementary Figure 12. Uncropped immunoblot images from Supplementary Figure 4. Red boxes show the cropping limits used to generate the final panels.

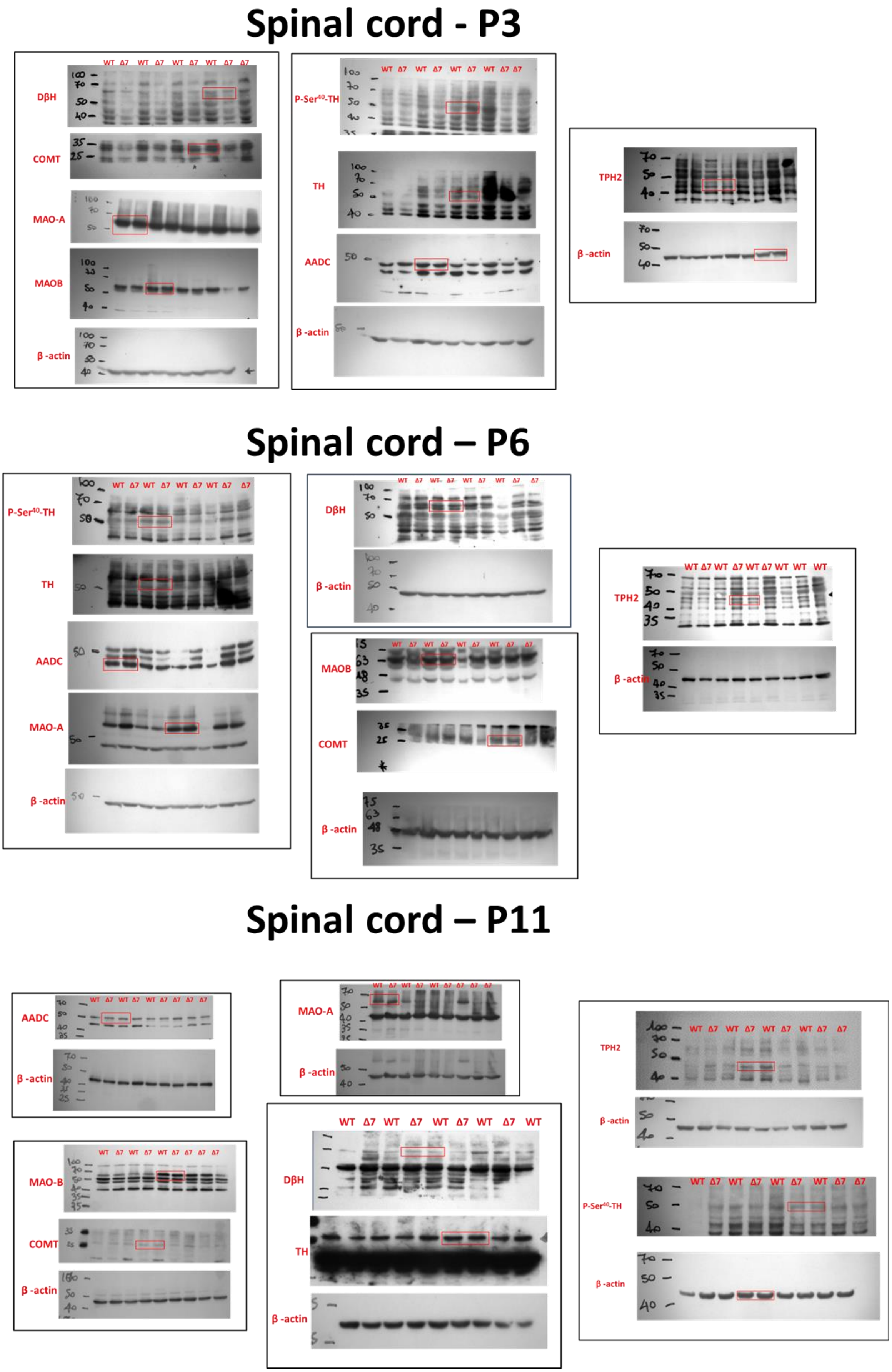

Supplementary Figure 13. Uncropped immunoblot images from Supplementary Figure 5. Red boxes show the cropping limits used to generate the final panels.

Supplementary Table 1. Biochemical pathways affected by SMN deficiency in the liver of *SMNΔ7* mice at P3.

Pathways	*Hits*	*Raw p*	*Holm p*	*FDR*	*Metabolites*
Aspartate Metabolism	6	0.00087884	0.079096	0.024209	Fumaric, Glutamine, Adenosine triphosphate,Acetic acid,L-Aspartic acid,L-Glutamic acid
Pyrimidine Metabolism	2	0.0011481	0.10218	0.024209	Glutamine,Adenosine triphosphate
Phenylacetate Metabolism	2	0.0011481	0.10218	0.024209	Glutamine, Adenosine triphosphate
Methionine Metabolism	6	0.0013008	0.11317	0.024209	Betaine,Glycine,Sarcosine, Adenosine triphosphate,L-Methionine,NAD
Betaine Metabolism	4	0.0013449	0.11566	0.024209	Betaine, Adenosine triphosphate,L-Methionine,NAD
Purine Metabolism	8	0.0019151	0.16278	0.028726	Fumaric, Glutamine, Adenosine triphosphate,Hypoxanthine,L-Aspartic acid,L-Glutamic acid,NAD,Glycine
Glycine and Serine Metabolism	10	0.0025765	0.021643	0.030639	Betaine,Glutamine,Creatine,Alanine,Glutamic acid, Ornithine,NAD,Adenosine triphospate,Sarcosine,Glycine
Warburg Effect	10	0.0027235	0.022605	0.030639	Citric acid, D-Glucose,Fumaric acid, L-Glutamic acid, L-Malic acid,L-Lactic acid,Succinic acid,Adenosine triphosphate,L-Glutamine,NAD
Urea Cycle	8	0.0031308	0.05672	0.031308	Fumaric acid, L-Glutamic acid, L-Alanine, L-Aspartic acid,Ornithine,Glutamine, Adenosine triphosphate,NAD
Nicotinate and Nicotinamide Metabolism	5	0.0061337	0.049683	0.054102	L-Glutamic acid, Adenosine triphosphate,L-Glutamine,NAD,Niacinamide
Spermidine and Spermine Biosynthesis	3	0.0069063	0.045251	0.054102	Methionine, Adenosine Triposhate,Ornithine
Amino Sugar Metabolism	4	0.0072135	0.046987	0.054102	Glutamine,Adenosine triphosphate,L-Glutamic acid,Acetic acid
Ammonia Recycling	6	0.01209	0.94306	0.080651	NAD, Glutamine, Adenosine triphosphate,Glycine,L-Aspartic acid,L-Glutamic acid
Glutamate Metabolism	9	0.012546	0.96602	0.080651	Glutathione,L-Glutamic acid,L-Alanine; L-Aspartic acid,Glutamine, Adenosine triphosphate
Sulfate/Sulfite Metabolism	2	0.022214	1	0.12724	Adenosine triphosphate,Glucose,Adenosine 3',5'-diphosphate
Sphingolipid Metabolism	3	0.022621	1	0.12724	Adenosine triphosphate,Glucose,Adenosine 3',5'-diphosphate
Glutathione Metabolism	6	0.026169	1	0.13854	Glutathione,L-Glutamic acid,L-Alanine; L-Aspartic acid,Glutamine, Adenosine triphosphate

Biochemical pathways were identified through Pathway Enrichment analysis in liver extracts of *SMNΔ7* and WT mice. Hits is the actually matched number of metabolites from the user uploaded data. *p* value is calculated from the enriched analysis. The false discovery rate (FDR) is the portion of false positives above the user-specified score threshold. Metabolites are the compounds corresponding to the Hits.

Supplementary Table 2. Biochemical pathways affected by SMN deficiency in the liver of *SMNΔ7* mice at P11.

Liver	Hits	Raw p	Holm p	FDR	Metabolites
Tyrosine metabolism	4	9.24E-15	3.70E-12	3.70E-12	Tyrosine, Fumarate, Pyruvate, Acetoacetate
Valine, leucine and isoleucine degradation	4	4.69E-12	1.83E-10	9.39E-11	Acetoacetate, Valine, Isoleucine, Leucine
Phenylalanine, tyrosine and tryptophan biosynthesis	2	7.25E-11	2.75E-09	7.25E-10	Phenylalanine, Tyrosine
Phenylalanine metabolism	2	7.25E-11	2.75E-09	7.25E-10	Phenylalanine, Tyrosine
Arginine biosynthesis	5	1.40E-10	5.03E-09	1.12E-09	Glutamate, Glutamine, Aspartate, Ornithine, Fumarate
Butanoate metabolism	4	1.85E-10	6.47E-09	1.23E-10	3-Hydroxybutanoate, Acetoacetate, Glutamate, Succinate
Alanine aspartate and glutamate metabolism	9	3.38E-10	1.15E-09	1.93E-09	Aspartate, Alanine, Glutamate, Glutamine, Citrate, Oxalacetate, Fumarate, Pyruvate, Succinate
Arginine and proline metabolism	4	6.92E-11	2.28E-08	3.46E-10	Creatine, Ornithine, Glutamate, Pyruvate
Citrate cycle (TCA cycle)	6	8.26E-10	2.64E-08	3.67E-09	Succinate, Oxalacetate, Malate, Citrate, Pyruvate,, Fumarate
Glutathione metabolism	6	2.41E-09	7.24E-08	8.77E-09	Gluthatione, NADP+, Glycine, Glutamate, Oxoproline, Ornithine
Aminoacyl-tRNA biosynthesis	13	1.62E-07	4.53E-07	4.98E-07	Phenylalanine, Glutamine, Glycine, Aspartate, Methionine, Valine, Alanine, lysine, Isoleucine, Threonine, Tyrosine, Glutamate
Synthesis and degradation of ketone bodies	2	5.84E-07	1.58E-05	1.67E-06	3-Hydroxybutanoate, Acetoacetate, Glutamate, Succinate
Nicotinate and nicotinamide metabolism	4	4.50E-06	1.17E-04	1.20E-05	Aspartate, NAD+, NADP+, Nicotinamide
Valine, leucine and isoleucine biosynthesis	4	1.99E-05	4.97E-04	4.97E-05	Threonine, Valine, Leucine, Isoleucine
Pyruvate metabolism	6	0.00010131	0.0023301	0.00022513	Oxalacetate, Pyruvate, Lactate, Succinate, Acetate, Fumarate

Biochemical pathways were identified through Pathway Enrichment analysis in liver extracts of *SMNΔ7* and WT mice. Hits is the actually matched number of metabolites from the user uploaded data. *p* value is calculated from the enriched analysis. The false discovery rate (FDR) is the portion of false positives above the user-specified score threshold. Metabolites are the compounds corresponding to the Hits.

Supplementary Table 3. Biochemical pathways affected by SMN deficiency in the brain *SMNΔ7* mice at P11.

Pathways	Hits	*p* value	FDR	Metabolites
Pyruvate Metabolism	2	7.15E-15	9.65E-05	L-lactic acid, Acetic acid
Carnitine Synthesis	2	3.19E-03	1.56E-02	Glycine, Succinate
Valine Leucine and Isoleucine Degradation	5	6.73E-02	3.03E-01	2-Hydroxyisovalerate, Isoleucine, Succinate, Leucine, Valine
Glycine and Serine Metabolism	5	1.27E-01	5.03E-01	Creatine, Glycine, Glutamate, Alanine, Threonine
Phenylalanine and Tyrosine Metabolism	2	1.60E-01	5.42E-02	Glutamate, Tyrosine
Tyrosine Metabolism	3	2.39E-01	7.58E-01	Tyrosine, Glutamate, Aspartate
Arginine and Proline Metabolism	5	0.00013013	0.00039039	Glutamate, Aspartate, Glycine, Succinate, Creatine
Warburg Effect	4	0.00017398	0.0004777	Glutamate, Lactate, Succinate, Glutamine
Glutamate Metabolism	7	0.00017693	0.0004777	Succinate, Alanine, Glutamine, Glycine, Gamma-Ammino-Butyric acid, Aspartate, Glutamate
Amino Sugar Metabolism	3	0.00034273	0.0008813	Acetate, Glutamate, Glutamine
Aspartate Metabolism	6	0.0026386	0.006195	Acetate, Inosinic acid, Glutamine, N-acetyl-L-aspartic acid, Aspartate, Glutamate
Purine Metabolism	5	0.031082	0.069935	Inosinic acid, Glutamine, Glycine, Glutamate, Aspartate

Biochemical pathways were identified through Pathway Enrichment analysis in brain extracts of *SMNΔ7* and WT mice. Hits is the actually matched number of metabolites from the user uploaded data. *p* value is calculated from the enriched analysis. The false discovery rate (FDR) is the portion of false positives above the user-specified score threshold. Metabolites are the compounds corresponding to the Hits.

Supplementary Table 4. Statistical analysis of monoamine levels in the brain and spinal cord of *SMNΔ7* mice during the ontogeny.

Molecules	Two-way ANOVA	Brain		Spinal cord	
		F (DFn. DFd)	*p value*	F (DFn. DFd)	*p value*
DA	Age x Genotype	F (2. 51) = 8.356	*p=0.0007*	F (2. 49) = 0.1691	*p=0.8449*
	Age	F (2. 51) = 132.3	*p<0.0001*	F (2. 49) = 184.5	*p<0.0001*
	Genotype	F (1. 51) = 11.52	*p=0.0013*	F (1. 49) = 0.4711	*p=0.4957*
NE	Age x Genotype	F (2. 51) = 3.247	*p=0.0471*	F (2. 49) = 2.923	*p=0.0632*
	Age	F (2. 51) = 1.357	*p=0.2666*	F (2. 49) = 2.893	*p=0.0649*
	Genotype	F (1. 51) = 15.71	*p=0.0002*	F (1. 49) = 12.31	*p=0.0010*
5-HT	Age x Genotype	F (2. 51) = 1.046	*p=0.3589*	F (2. 49) = 0.2907	*p=0.7490*
	Age	F (2. 51) = 14.23	*p<0.0001*	F (2. 49) = 42.06	*p<0.0001*
	Genotype	F (1. 51) = 7.495	*p=0.0085*	F (1. 49) = 0.8205	*p=0.3695*
L-DOPA	Age x Genotype	F (2. 51) = 0.444	*p=0.6439*	F (2. 49) = 0.04335	*p=0.9576*
	Age	F (2. 51) = 10.7	*p=0.0001*	F (2. 49) = 99.01	*p<0.0001*
	Genotype	F (1. 51) = 0.129	*p=0.7210*	F (1. 49) = 1.408	*p=0.2411*
DOPAC	Age x Genotype	F (2. 51) = 0.6957	*p=0.5034*	F (2. 49) = 4.593	*p=0.0149*
	Age	F (2. 51) = 33.59	*p<0.0001*	F (2. 49) = 35.24	*p<0.0001*
	Genotype	F (1. 51) = 1.26	*p=0.2670*	F (1. 49) = 2.289	*p=0.1367*
HVA	Age x Genotype	F (2. 51) = 0.1919	*p=0.8260*	F (2. 49) = 1.696	*p=0.1940*
	Age	F (2. 51) = 43.39	*p<0.0001*	F (2. 49) = 51.48	*p<0.0001*
	Genotype	F (1. 51) = 0.09904	*p=0.7543*	F (1. 49) = 0.6918	*p=0.4096*
5-HIAA	Age x Genotype	F (2. 51) = 3.161	*p=0.0508*	F (2. 49) = 16.09	*p<0.0001*
	Age	F (2. 51) = 3.462	*p=0.0389*	F (2. 49) = 53.98	*p<0.0001*
	Genotype	F (1. 51) = 15.83	*p=0.0002*	F (1. 49) = 19.44	*p<0.0001*
DA/L-DOPA	Age x Genotype	F (2. 51) = 0.4216	*p=0.6582*	F (2. 45) = 3.459	*p=0.0400*
	Age	F (2. 51) = 11.72	*p<0.0001*	F (2. 45) = 1.046	*p=0.3598*
	Genotype	F (1. 51) = 1.491	*p=0.2276*	F (1. 45) = 0.9205	*p=0.3425*
DOPAC/DA	Age x Genotype	F (2. 51) = 0.4177	*p=0.6608*	F (2. 49) = 0.5934	*p=0.5564*
	Age	F (2. 51) = 13.57	*p<0.0001*	F (2. 49) = 30.99	*p<0.0001*
	Genotype	F (1. 51) = 0.4084	*p=0.5256*	F (1. 49) = 0.2302	*p=0.6335*
5-HIAA/5-HT	Age x Genotype	F (2. 51) = 2.581	*p=0.0855*	F (2. 49) = 5.477	*p=0.0071*
	Age	F (2. 51) = 4.885	*p=0.0115*	F (2. 49) = 6.588	*p=0.0029*
	Genotype	F (1. 51) = 2.421	*p=0.1259*	F (1. 49) = 15.13	*p=0.0003*

Statistical analyses were performed by two-way ANOVA considering age (post-natal day 3 *vs* day 6 *vs* day 11) and genotype (WT *vs* *SMNΔ7*) as factors.

Supplementary Table 5. Demographic and clinical characteristics of naïve SMA1 patients and healthy control subjects enrolled in NMR analysis.

	Control (n=3)	SMA1 (n=3)
	Median[range]	Median[range]
Sex (female/male)	2 / 1	2 / 1
Age (years)	11[4;12]	3.47[0.37;3.6]
BMI	-	13.46[11.57;14.87]
SMN2 copy (2/3/4)	-	3/0/0
CHOP-INTEND	-	6[0;27]
Gastrostomy (No/Yes)	-	1 / 2
NIV (No / Yes)	-	2 / 1
Tracheostomy (No/Yes)	-	2 / 1
Total CSF proteins (µg/µL)	0.12[0.092;0.167]	0.137[0.13;0.23]
CSF pH	8.7[8.67;8.8]	8.53[8.32;8.6]

Values are expressed as median [min; max]. For sex, gastrostomy, NIV and tracheostomy, the number of subjects is indicated. Abbreviations: BMI = Body Mass Index; CHOP-INTEND = Children's Hospital of Philadelphia Infant Test of Neuromuscular Disorders; NIV= Non-invasive ventilation; CSF = cerebrospinal fluid.

Supplementary Table 6. Demographic and clinical characteristics of naïve SMA1, SMA2 and SMA3 patients enrolled in HPLC analysis.

Demographic and clinical information	SMA1 (n=12)	SMA2 (n=10)	SMA3 (n=11)
	Median[range]	Median[range]	Median[range]
Sex (female/male)	7/5	5/5	11/0
SMN2 copy (2/3/4)	11/1/0	1/9	2/7/2
Age (years)	3.04[0.12;5.74]	3.96[1.22;13.3]	12.64[2.29;17.98]
BMI	13.19[11.56;14.86]	17.18[13.78;20.4] (n=8)	19.71[10.74;26.56] (n=10)
CHOP-INTEND	10.5[0;52]	-	-
HFMSE	-	8.5[2;41]	45[15;62]
Gastrostomy (No/Yes)	2/10	10/0	11/0
NIV (No/Yes)	6/6	7/3	10/1
Tracheostomy (No/Yes)	8/4	10/0	11/0

Values are expressed as median [min; max]. For sex, gastrostomy, NIV and tracheostomy, the number of subjects is indicated. Abbreviations: BMI = Body Mass Index; CHOP-INTEND = Children's Hospital of Philadelphia Infant Test of Neuromuscular Disorders; HFMSE = Hammersmith Functional Motor Scale Expanded; NIV= Non-invasive ventilation; CSF = cerebrospinal fluid.

Supplementary Table 7. Monoamine levels (expressed as nM) at T0 compared with T1 or T2 of Nusinersen treatment within each SMA1, SMA2 or SMA3 group.

	**SMA1**					**SMA2**					**SMA3**				
	T0 (N=12)	T1 (N=12)	T2 (N=10)	T0 *vs* T1	T0 *vs* T2	T0 (N=10)	T1 (N=6)	T2 (N=10)	T0 *vs* T1	T0 *vs* T2	T0 (N=11)	T1 (N=11)	T2 (N=11)	T0 *vs* T1	T0 *vs* T2
Metabolites	Median [range]			*p* value	*p* value	Median [range]			*p* value	*p* value	Median [range]			*p* value	*p* value
NE (nM)	9.54[0.1;16.64]	13.91[2.73;44]	13.96[1.23;56.78]	**0.006****	**0.005****	9.57[4.83;30.74]	11.97[4.94;18.14]	10.16[2.25;37.3]	0.916	0.721	9.39[2;62.66]	13.08[1.79;21.2]	10.6[2.58;20.98]	0.286	0.533
5-HT (nM)	186.69[45.76;251.01]	163.87[94.58;243.57]	187.83[40.26;269.03]	0.875	0.202	177.5[61.53;224.88]	149.7[103.37;186.18]	185.41[36.3;215.3]	0.463	0.721	164.52[51.32;298.42]	178.9[42.52;229.02]	142.64[34.56;271.87]	0.722	0.929
5-HIAA (nM)	240.8[109.22;747.43]	245.93[137.5;1010.65]	244.4[74.2;1014.3]	0.084	0.646	186.07[73.65;347.46]	169.36[125.64;276.91]	253.62[36.64;313.48]	0.463	0.721	184.91[21.97;287.86]	165.1[55.58;265.52]	157.04[30.86;335.72]	0.789	0.789

Values are expressed as median [min; max]. Statistical analyses were performed by non-parametric Wilcoxon matched-pairs signed ranks. Significant *p* values are also marked with asterisks (***p*<0.01).

Supplementary Table 8. Correlation analysis between CSF monoamine levels and age, BMI or motor outcomes of SMA1, SMA2 and SMA3 patients at T0 and T2 of Nusinersen therapy.

Patients	Therapy phase	Parameter	Statistics	NE	5-HT	5-HIAA
SMA1	T0	Age (months)	r	-0,315	0,028	0,224
			p value	0,319	0,931	0,484
			N	12	12	12
		BMI	r	0,056	0,357	0,196
			p value	0,863	0,255	0,542
			N	12	12	12
		CHOP-INTEND	r	-0,109	0,298	0,175
			p value	0,736	0,346	0,585
			N	12	12	12
		ΔCHOP-INTEND	r	0,419	0,160	-0,406
			p value	0,229	0,659	0,244
			N	10	10	10
	T2	Age (months)	r	-0,079	0,030	0,006
			p value	0,829	0,934	0,987
			N	10	10	10
		BMI	r	-0,067	0,233	0,283
			p value	0,865	0,546	0,460
			N	9	9	9
		CHOP-INTEND	r	-0,432	0,182	0,207
			p value	0,213	0,614	0,567
			N	10	10	10
		ΔCHOP-INTEND	r	-0,246	0,123	0,025
			p value	0,493	0,735	0,946
			N	10	10	10
SMA2	T0	Age (months)	r	0,200	0,309	0,406
			p value	0,580	0,385	0,244
			N	10	10	10
		BMI	r	-0,333	0,548	0,381
			p value	0,420	0,160	0,352
			N	8	8	8
		HFMSE	r	-0,280	0,134	-0,177
			p value	0,432	0,712	0,625
			N	10	10	10
		ΔHFMSE	r	0,037	-0,092	-0,166
			p value	0,919	0,800	0,646
			N	10	10	10
	T2	Age (months)	r	0,115	0,624	0,539
			p value	0,751	0,054	0,108
			N	10	10	10
		BMI	r	-0,250	0,000	-0,036
			p value	0,589	1,000	0,939

			N	7	7	7
		HFMSE	r	-0,030	-0,406	-0,491
			p value	0,934	0,244	0,150
			N	10	10	10
		ΔHFMSE	r	-0,080	-0,375	-0,308
			p value	0,826	0,285	0,387
			N	10	10	10
SMA3	T0	Age (months)	r	0,155	-0,318	-0,364
			p value	0,650	0,340	0,272
			N	11	11	11
		BMI	r	-0,248	-0,188	-0,503
			p value	0,489	0,603	0,138
			N	10	10	10
		HFMSE	r	0,542	-0,228	-0,009
			p value	0,085	0,501	0,979
			N	11	11	11
		ΔHFMSE	r	-0,267	-0,051	-0,207
			p value	0,428	0,883	0,542
			N	11	11	11
	T2	Age (months)	r	-0,297	-0,091	0,036
			p value	0,405	0,790	0,915
			N	10	11	11
		BMI	r	-0,321	-0,055	0,236
			p value	0,365	0,873	0,484
			N	10	11	11
		HFMSE	r	0,236	0,036	-0,218
			p value	0,511	0,915	0,519
			N	10	11	11
		ΔHFMSE	r	0,170	-0,239	-0,290
			p value	0,617	0,479	0,388
			N	11	11	11

CSF monoamine levels and demographic/clinical parameters were assessed at the time of the first (T0, baseline) and sixth (T2, maintenance phase) injection of Nusinersen. Motor outcomes in SMA1 patients were evaluated by CHOP-INTEND score, while in SMA2 and SMA3 patients by HFMSE. Statistical analysis was performed within each treatment group by Spearman's correlations. All *p* values were not significant after correction with Benjamini-Hochberg multiple comparisons. ΔCHOP-INTEND or ΔHFMSE represent the difference of the respective motor outcome scores between T2 and T0. Abbreviations: CHOP-INTEND = Children’s Hospital of Philadelphia Infant Test of Neuromuscular Disorders; HFMSE = Hammersmith Functional Motor Scale Expanded; BMI = Body Mass Index.

Supplementary Table 9. Clinical characteristics of SMA1, SMA2 and SMA3 patients enrolled in the study at the fourth (T1, loading phase) and sixth (T2, maintenance phase) injection of Nusinersen.

		CHOP-INTEND		HFMSE		Gastrostomy		NIV		Tracheostomy		CSF total proteins (µg/µL)	CSF pH
	Therapy phase	n	Median[range]	n	Median[range]	No	Yes	No	Yes	No	Yes	Median[range]	Median[range]
SMA1	**T0**	12	10.5[0;52]			2	10	6	6	8	4	0.17[0.1;0.49]	8.73[8.32;8.99]
	**T1**	11	16[0;64]			2	10	5	7	8	4	0.20[0.08;0.41]	8.73[8.05;9.17]
	**T2**	10	15.5[0;64]			1	9	4	6	7	3	0.20[0.07;0.54]	8.88[7.74;9.09]
SMA2	**T0**			10	8.5[2;41]	10	0	7	3	10	0	0.20[0.13;0.98]	8.83[7.6;9.29]
	**T1**			6	7.5[2;23]	6	0	5	1	6	0	0.20[0.12;0.33]	8.79[8.32;8.92]
	**T2**			10	12.5[2;57]	10	0	7	3	10	0	0.20[0.13;0.31]	8.81[8.26;9.06]
SMA3	**T0**			11	45[15;62]	11	0	10	1	11	0	0.23[0.1;0.38]	8.92[8.44;9.27]
	**T1**			9	39[17;62]	11	0	11	0	11	0	0.23[0.1;0.41]	8.94[7.87;9.44]
	**T2**			11	48[14;62]	11	0	10	1	11	0	0.33[0.11;0.61]	8.98[8.13;9.34]

Values are shown as median [min; max]. Statistical analyses of total CSF proteins (expressed as µg/µL) and CSF pH were performed by Kruskal-Wallis test. Abbreviations: CHOP-INTEND = Children's Hospital of Philadelphia Infant Test of Neuromuscular Disorders; HFMSE = Hammersmith Functional Motor Scale Expanded; NIV= non-invasive ventilation; CSF = Cerebrospinal fluid.
