# Supplementary material for: SMN deficiency perturbs monoamine neurotransmitter metabolism in spinal muscular atrophy
Source: Commun Biol. 2023 Nov 13;6:1155. doi: 10.1038/s42003-023-05543-1 (PMC10643621; doi:10.1038/s42003-023-05543-1)
Supplement: Supplementary file 3 — Description of Additional Supplementary Files [file 42003_2023_5543_MOESM3_ESM.pdf]

Description of Additional Supplementary Files

File name: Supplementary Data 1
Description: Excel file including the raw data of Figure 4 and Supplementary Figure 2.

File name: Supplementary Data 2
Description: Excel file including the raw data of Figures 5-7 and Supplementary Figures 3-7

File name: Supplementary Data 3
Description: Excel file including the raw data of Figure 9 and Supplementary Figure 8
