# Supplementary material for: SMN deficiency perturbs monoamine neurotransmitter metabolism in spinal muscular atrophy
Source: Commun Biol. 2023 Nov 13;6:1155. doi: 10.1038/s42003-023-05543-1 (PMC10643621; doi:10.1038/s42003-023-05543-1)
Supplement: Supplementary file 7 — Reporting Summary [file 42003_2023_5543_MOESM7_ESM.pdf]

nature portfolio

Corresponding author(s): COMMSBIO-23-1500A

Last updated by author(s): Oct 30, 2023

Reporting Summary

Nature Portfolio wishes to improve the reproducibility of the work that we publish. This form provides structure for consistency and transparency in reporting. For further information on Nature Portfolio policies, see our Editorial Policies and the Editorial Policy Checklist.

Statistics

For all statistical analyses, confirm that the following items are present in the figure legend, table legend, main text, or Methods section.

n/a	Confirmed	
☐	☒	The exact sample size (*n*) for each experimental group/condition, given as a discrete number and unit of measurement
☐	☒	A statement on whether measurements were taken from distinct samples or whether the same sample was measured repeatedly
☐	☒	The statistical test(s) used AND whether they are one- or two-sided<br>*Only common tests should be described solely by name; describe more complex techniques in the Methods section.*
☐	☒	A description of all covariates tested
☐	☒	A description of any assumptions or corrections, such as tests of normality and adjustment for multiple comparisons
☐	☒	A full description of the statistical parameters including central tendency (e.g. means) or other basic estimates (e.g. regression coefficient) AND variation (e.g. standard deviation) or associated estimates of uncertainty (e.g. confidence intervals)
☐	☒	For null hypothesis testing, the test statistic (e.g. *F*, *t*, *r*) with confidence intervals, effect sizes, degrees of freedom and *P* value noted<br>*Give P values as exact values whenever suitable.*
☒	☐	For Bayesian analysis, information on the choice of priors and Markov chain Monte Carlo settings
☒	☐	For hierarchical and complex designs, identification of the appropriate level for tests and full reporting of outcomes
☒	☐	Estimates of effect sizes (e.g. Cohen's *d*, Pearson's *r*), indicating how they were calculated

Our web collection on statistics for biologists contains articles on many of the points above.

Software and code

Policy information about availability of computer code

Data collection	All software used in this study for data collection are either commercially available or open source.
Data analysis	All software used in this study for data analysis are either commercially available or open source.

For manuscripts utilizing custom algorithms or software that are central to the research but not yet described in published literature, software must be made available to editors and reviewers. We strongly encourage code deposition in a community repository (e.g. GitHub). See the Nature Portfolio guidelines for submitting code & software for further information.

Data

Policy information about availability of data

All manuscripts must include a data availability statement. This statement should provide the following information, where applicable:

- Accession codes, unique identifiers, or web links for publicly available datasets
- A description of any restrictions on data availability
- For clinical datasets or third party data, please ensure that the statement adheres to our policy

The datasets generated during and/or analyzed during the current study are available as Supplementary Material (Supplementary Data 1-3). NMR data have been deposited to the EMBL-EBI MetaboLights database (DOI: 10.1093/nar/gkz1019, PMID:31691833) with the identifier MTBLS8784.

Research involving human participants, their data, or biological material

Policy information about studies with human participants or human data. See also policy information about sex, gender (identity/presentation), and sexual orientation and race, ethnicity and racism.

Reporting on sex and gender	Sex or gender was determined based on self-reporting.
Reporting on race, ethnicity, or other socially relevant groupings	No social relevant categorization was considered.
Population characteristics	All participants are in pediatric age.
Recruitment	All patients were clinically diagnosed and genetically confirmed, and SMN2 copy number was also determined.
Ethics oversight	The study was approved by the local Ethics Committees.

Note that full information on the approval of the study protocol must also be provided in the manuscript.

Field-specific reporting

Please select the one below that is the best fit for your research. If you are not sure, read the appropriate sections before making your selection.

☒ Life sciences ☐ Behavioural & social sciences ☐ Ecological, evolutionary & environmental sciences

For a reference copy of the document with all sections, see nature.com/documents/nr-reporting-summary-flat.pdf

Life sciences study design

All studies must disclose on these points even when the disclosure is negative.

Sample size	No sample size evaluations were performed.
Data exclusions	There are data exclusions for technical reasons or exhausted samples.
Replication	All attempts to replicate experiments were successful.
Randomization	Randomly selected samples were allocated into experimental groups.
Blinding	Blinding was not implemented in this study.

Reporting for specific materials, systems and methods

We require information from authors about some types of materials, experimental systems and methods used in many studies. Here, indicate whether each material, system or method listed is relevant to your study. If you are not sure if a list item applies to your research, read the appropriate section before selecting a response.

Materials & experimental systems

n/a	Involved in the study	
☐	☒	Antibodies
☒	☐	Eukaryotic cell lines
☒	☐	Palaeontology and archaeology
☐	☒	Animals and other organisms
☒	☐	Clinical data
☒	☐	Dual use research of concern
☒	☐	Plants

Methods

n/a	Involved in the study	
☒	☐	ChIP-seq
☒	☐	Flow cytometry
☒	☐	MRI-based neuroimaging

Antibodies

Antibodies used	For western blotting: anti-TH (mouse monoclonal antibody, 1:1000; Millipore, Milan, Italy); anti-P-TH-Ser40 (rabbit polyclonal antibody, 1:1000; Cell Signaling, Danvers, MA, USA); anti-AADC (rabbit polyclonal antibody, 1:1000; Novus Biological, Centennial, CO, USA); anti-DβH (rabbit polyclonal antibody, 1:1000; Novus Biological); anti-TPH2 (mouse monoclonal antibody, 1:1000; Abcam, Boston; MA, USA); anti-MAO-A (rabbit polyclonal antibody, 1:1000; Abcam); anti-MAO-B (rabbit polyclonal antibody, 1:1000; Abcam);

	anti-COMT (rabbit polyclonal antibody, 1:1000; Abcam); anti-PAH (rabbit polyclonal antibody, 1:500; Novus Biological).<br><br>For immunostaining: anti-AADC (1:100, Abcam); anti-TH (1:250, Millipore); anti-TPH2 (1:250, Abcam)
Validation	All the anitbodies used in this study are commercial and validated.

Animals and other research organisms

Policy information about studies involving animals; ARRIVE guidelines recommended for reporting animal research, and Sex and Gender in Research

Laboratory animals	- Smn+/+ (wild type) and Smn-/- (SMNΔ7) mice used in this study derive from SMN2+/-;SMNΔ7+/-;Smn+/- mice, heterozygous and healthy carrier for Smn gene mutation (purchased from Jackson Laboratory stock number 005025; Jackson Laboratories, Bar Harbor, ME, USA)<br><br>- Transgenic mice B6SJLTgN(SOD1G93A)1Gur (overexpressing human SOD1, containing the Gly93Ala (G93A) mutation, The Jackson Laboratory, stock number 002726) used in this study derive from male transgenic mice and naïve (B6xSJL/J)F1 females (wild type) (The Jackson Laboratory, stock number 100012).
Wild animals	The study did not involve wild animals.
Reporting on sex	Male mice were used in this study.
Field-collected samples	The study did not involve animals collected on the field.
Ethics oversight	Experiments were performed according to the international guidelines for animal research and approved by the Animal Care Committee of the University of Naples Federico II, Italy, and Ministry of Health, Italy.

Note that full information on the approval of the study protocol must also be provided in the manuscript.
